# Where Is Equity in HiAP? Comment on "A Realist Explanatory Case Study Investigating How Common Goals, Leadership, and Committed Staff Facilitate Health in All Policies Implementation in the Municipality of Kuopio, Finland"

**DOI:** 10.34172/ijhpm.2023.7611

**Published:** 2023-04-10

**Authors:** Michelle Amri, Jesse B. Bump

**Affiliations:** ^1^Takemi Program in International Health, Harvard T.H. Chan School of Public Health, Boston, MA, USA; ^2^Bergen Centre for Ethics and Priority Setting, University of Bergen, Bergen, Norway

**Keywords:** Health in All Policies, Healthy Public Policy, Multisectoral Health Policy, Intersectoral Health Policy, Global Health, Health Policy

## Abstract

Health equity is no longer a central feature of Health in All Policies (HiAP) approaches despite its presence in select definitions of HiAP. In other words, HiAP is not just about considering health, but also health equity. But as HiAP has become more mainstream, its success around health equity has been muted and largely non-existent. Given the normative underpinning and centrality of equity in HiAP, equity should be better considered in HiAP and particularly when considering what ‘successful’ implementation may look like. Raising health on the radar of policy-makers is not mutually exclusive from considering equity. Taking an incremental approach to considering equity in HiAP can yield positive results. This article discusses these ideas and presents potential actions to restore HiAP’s once central equity objectives, which include: seeking synergies focused on health equity with those who hold different convictions, both in terms of goals and measures of success; considering the conditions that allow HiAP to be fostered, such as good governance; and drawing on research on HiAP and other multisectoral approaches.

## Introduction

 The centrality of health in public policy-making has been recognized by many researchers and practitioners in the health sector. One particularly prominent approach is Health in All Policies (HiAP), which is “an approach to public policy that systematically takes into account the health implications of decisions, seeks synergies, and avoids harmful health impacts to improve population health and health equity.”^1^ This would imply assessing the health and health equity implications of infrastructure projects or agricultural policy, for example. Those working in the health sector have long recognized the vulnerability of health to decisions in other sectors. For example, promoting tobacco in one sector, which results in negative health outcomes, particularly for certain segments of society. And similarly, it is known that infrastructure is linked to activity patterns, pollution, and many other things, that again, have disproportionate impacts. But the health sector is unable to influence much of these things on its own. Thus, HiAP was created with the aim of sensitizing those in other sectors to health and health equity. As HiAP has become more mainstream, its success in raising health on the radar of policy-makers in traditionally non-health sectors has been realized in many settings, while its success around health equity has been muted and largely non-existent.

## Where Is Equity in HiAP?

 While HiAP is a relatively well-developed area of inquiry, ongoing research and conversations do not always center efforts on health equity, despite equity arguably being key in HiAP. This focus on equity is evident in select definitions of HiAP where “equity” is emphasized, such as through noting that HiAP is “about integrated governance which promotes health and *equity* objectives and at the same time achieves mutual benefits for partnering sectors”^2^ (emphasis added). Or, given that “promoting health and equity” is noted as one of the five key elements of HiAP and that HiAP “…seeks to institutionalize considerations of health, equity, and sustainability as a standard part of decision-making processes across a broad array of sectors.” ^3^ Thus, HiAP is not just about considering health, but also health equity. But policy-makers taking a HiAP approach tend to focus on having various sectors systematically consider the health implications of decisions (eg, what are the health implications of the construction of a major road?). This process of just considering health neglects equity (eg, in what ways are different populations impacted from this development?). It is not that equity does not matter, but rather, addressing health equity is arguably more difficult than simply addressing health outcomes. This may be for a host of different reasons, including different conceptualizations of health equity by different stakeholders given that it a normative term and that considering equity can entail a redistribution of resources and disrupting the status quo, which is difficult politically. Therefore, focusing on health outcomes as opposed to improving health equity, while more straightforward, does not address health inequities across populations and is a missed opportunity for more impactful and just action. For instance, while the development of a public park may be beneficial for increasing physical activity among children, consideration should be afforded to where these parks would have greater impact and are more needed. Similarly, in undertaking transportation projects, it is not enough to consider air quality, congestion, and greenhouse gas emissions, but consideration should also be afforded to who can reasonably access public transportation options, whether in terms of location or cost.

 Although one may argue that it is easier to strive to complete one project without any equity-focused targets than simply completing a project — because equity will never fully be accomplished as a goal — we feel these do not need to be mutually exclusive. Consider the example of the park; considering equity in terms of where the park should be located does not necessarily lead to a more convoluted process or the desire to establish a park being abandoned. Further, even if it was the case that equity-focused initiatives are less likely to be accepted or acted on, we feel that taking an incremental approach towards improving health equity is needed. Health equity can be improved in small incremental steps through focusing on achievable policy targets.

 Given the centrality of equity in HiAP, consideration should be afforded to equity in assessing HiAP and particularly when thinking about what ‘successful’ implementation may look like. When considering what ‘successful’ implementation looks like, drawing on Guglielmin and colleagues’ work as an example, they asked the sub-question, “What are the underlying mechanisms facilitating successful implementation?” They defined successful implementation “as positive policy implementation outcomes including acceptability and feasibility of implementation across parties involved, and sustainability of the HiAP implementation process (eg, completion of a HiAP intervention activity).”^4^ We appreciate this transparent approach to defining what success means to the authors. While we agree that it is incredibly important for the approach to be accepted and efforts sustained, naturally, we also feel it is important that considerations of success reflect the root of what HiAP seeks to do: improve population well-being and health equity. Assessing successful HiAP implementation should therefore also entail considering how both health and health equity are embedded in the process. For instance, assessing if discussions on health and health equity were taken with both health- and non-health-focused policy-makers. A finding from Guglielmin and colleagues’ work also points to the importance of this through not only noting the significance of communication and collaboration across sectors for common goals, but also the observation that once some non-health sector employees realized the role their sector played in influencing health and well-being, they incorporated health and well-being in their planning and actions.^4^ While HiAP program evaluation frameworks can be used to assess policy processes and outcomes, as discussed by Lawless et al^5^ in the case of South Australia, this may be drawn on as one potential avenue to consider how equity can be better reflected in HiAP policy processes and outcomes.

## Maximize Equity Under HiAP

 With HiAP’s focus shifting away from health equity, HiAP’s equity objectives that were arguably once central now need to be restored. Because HiAP is a normative conviction (ie, health should be prioritized and if consideration is afforded to health equity, that equity should be sought), those holding different convictions should seek equity-focused synergies to strive for the major goals of HiAP. To illustrate, we want to reiterate how crucial one of Guglielmin and colleagues’ main findings is around the importance of common goals, which also emerges in other HiAP scholarship. This has also come through in findings from a recent umbrella review that looks across various intersectoral and multisectoral policy approaches, including HiAP, but also One Health.^6^ Having a shared vision or common goals — the latter term being preferred by the participants in Guglielmin and colleagues’ study as they note — can work not only to enable intersectoral and multisectoral action, but also its absence was widely cited as a barrier to intersectoral and multisectoral health policy.^6^ A first step to refocusing on health equity and seeking related synergies may to be ignite conversation across stakeholders, which can be accomplished by drawing on scholarship investigating approaches to health equity^7,8^ to yield some points for discussion. Evidently, there can be no expectation that those working in traditionally non-health sectors will be well-acquainted with the concept of health; the social, political, commercial, and other determinants of health; health equity; and other related concepts and terms, given that the aforementioned work notes how approaches to health equity can vary even by prominent health actors, such as the World Health Organization (WHO).^7,8^

 Given differing convictions of different stakeholders, alignment should be sought not just in goals (ie, common health and non-health goals), but in how these goals are assessed with respect to equity. This is important given that policy-makers are accountable to their employers, whether it be a ministry or department, and their priorities and measures of success. For instance, it is simply not enough for champions of health to seek common goals such as redirecting sin taxes to health promotion activities when a ministry of finance may be focused on taking austerity measures that will negatively impact some segments of the population more than others. Instead, HiAP champions must seek to establish tangible common indicators of success that seek to bring about improved health and well-being outside of a more traditional definition and focus on emphasizing equitable outcomes. We recognize that this is a highly political task, both in terms of defining what success means and in determining how to assess how health and health equity have been embedded. This is particularly challenging when considering the different ways health equity and equity more broadly are understood and how associated goals can differ (targeting of sub-populations, ensuring access to services for all, providing certain resources to select groups or for all, etc). However, we believe it is important to be transparent about this as we advance collectively, given that these discussions can transcend to policy-makers, particularly those with different foci and goals.

 Emphasis also needs to be placed on creating the conditions where HiAP can be fostered — particularly with respect to good governance, however that may be defined.^9^ In fact, the absence of governance mechanisms for HiAP was identified as being a key finding across a global review of HiAP in 41 countries.^10^ There is nothing inherently different with Kuopio when compared to other cities. But the case of Kuopio demonstrates what a social contract may look like and how attention should also be directed to the conditions that allow HiAP to flourish. Then, approaches to HiAP can be strengthened and sustained and improvements to health, well-being, and equity can be sought.

 In acting on these challenges, research on HiAP and other multisectoral approaches may be beneficial in guiding the thinking around how to best proceed with aligning convictions around health and equity and clarifying measures of success. For instance, drawing on the work of Guglielmin et al and the successes in Kuopio, the importance of common goals between sectors and local leadership and committed staff is noted, allowing champions of HiAP to ensure they have these essential ingredients early on. And similarly, we can learn from Kuopio in terms of pitfalls to avoid. Take the establishment of intersectoral committees, and in the case of Kuopio, the well-being committee, as an example. As Guglielmin et al^4^ note, the strategy of an intersectoral committee does not necessarily entail positive benefits. In their work, they found the mechanism by which gains are realized is the discussion between personnel across various sectors, rather than simply having such a committee. And additionally, noting that such committees have been found be ineffective in some Danish cities.^11^ Therefore, champions of HiAP should seek to draw on evidence around HiAP to inform action, while centering normative commitments to equity. It is not that evidence and principles are at odds, but rather these can be merged for improved action through a more fulsome consideration of health equity. What are the facilitators and barriers to HiAP implementation across numerous ways of working multisectorally?^12^ How can health and health equity be centred in HiAP approaches? Can health equity be more greatly considered through a synergized Healthy Cities and HiAP approach?^13^ What is it that leads to better HiAP implementation? And so forth. The work of Guglielmin et al provides one piece of the puzzle for this latter question and can help us understand the conditions in Kuopio that supported HiAP uptake.

## Conclusion

 With the heightened prominence of HiAP and multisectoral ways of working, including calls from high-ranking policy-makers for HiAP to be undertaken^14^ and policy strategies focused on HiAP adopted, such as in Burundi,^15^ we are at an opportune time to meaningfully reflect and tread deliberately. HiAP provides numerous benefits but only when it used deliberately and advantageously, and attention is paid to fostering the conditions in which HiAP can be ‘successfully’ deployed.

 With health equity being absent from much of the work on HiAP, there is a cost for the populations that HiAP seeks to reach. Although health equity has different meanings to different individuals and full equity is likely impossible to achieve, this does not mean that equity should not be sought. This is particularly important when considering the aim of HiAP to manage all forces in health — hence its title. Without equity, HiAP approaches are in vain.

## Ethical issues

 Not applicable.

## Competing interests

 Authors declare that they have no competing interests.

## Authors’ contributions

**Conceptualization:** Michelle Amri, Jesse B. Bump.

**Writing–original draft: **Michelle Amri.

**Writing–review & editing:** Michelle Amri, Jesse B. Bump.

## Funding

 This work was supported by a Social Sciences and Humanities Research Council Postdoctoral Fellowship held by MA. This funding body had no influence on the design of the study; collection, analysis, and interpretation of data; and in writing or submitting the manuscript.

## References

[1] Pan American Health Organization. About Health in All Policies. https://www3.paho.org/hq/index.php?option=com_content&view=article&id=9360:2014-about-health-all-policies&Itemid=40177&lang=en.

[2] World Health Organization. Promoting Health in All Policies and Intersectoral Action Capacities. https://www.who.int/activities/promoting-health-in-all-policies-and-intersectoral-action-capacities.

[3] Rudolph L, Caplan J, Ben-Moshe K, Dillon L. Health in All Policies: A Guide for State and Local Governments. Washington, DC: American Public Health Association, Public Health Institute; 2013.

[4] Guglielmin M, Shankardass K, Bayoumi A, O’Campo P, Kokkinen L, Muntaner C (2022). A realist explanatory case study investigating how common goals, leadership, and committed staff facilitate Health in All Policies implementation in the municipality of Kuopio, Finland. Int J Health Policy Manag.

[5] Lawless A, Baum F, Delany-Crowe T (2018). Developing a framework for a program theory-based approach to evaluating policy processes and outcomes: Health in All Policies in South Australia. Int J Health Policy Manag.

[6] Amri M, Chatur A, O’Campo P (2022). An umbrella review of intersectoral and multisectoral approaches to health policy. Soc Sci Med.

[7] Amri MM, Jessiman-Perreault G, Siddiqi A, O’Campo P, Enright T, Di Ruggiero E (2021). Scoping review of the World Health Organization’s underlying equity discourses: apparent ambiguities, inadequacy, and contradictions. Int J Equity Health.

[8] Amri M, O’Campo P, Enright T, Siddiqi A, Di Ruggiero E, Bump JB (2022). Probing key informants’ views of health equity within the World Health Organization’s Urban HEART initiative. BMC Public Health.

[9] Lee K, Kamradt-Scott A (2014). The multiple meanings of global health governance: a call for conceptual clarity. Global Health.

[10] Ortenzi F, Marten R, Valentine NB, Kwamie A, Rasanathan K (2022). Whole of government and whole of society approaches: call for further research to improve population health and health equity. BMJ Glob Health.

[11] Holt DH, Carey G, Rod MH (2018). Time to dismiss the idea of a structural fix within government? An analysis of intersectoral action for health in Danish municipalities. Scand J Public Health.

[12] Amri M, Chatur A, O’Campo P (2022). Intersectoral and multisectoral approaches to health policy: an umbrella review protocol. Health Res Policy Syst.

[13] Amri M (2022). Healthy governance for cities: synergizing Health in All Policies (HiAP) and healthy cities approaches. J Urban Health.

[14] Tumusiime P, Karamagi H, Titi-Ofei R (2020). Building health system resilience in the context of primary health care revitalization for attainment of UHC: proceedings from the Fifth Health Sector Directors’ Policy and Planning Meeting for the WHO African Region. BMC Proc.

[15] Karamagi H, Titi-Ofei R, Amri M (2022). Cross country lessons sharing on practices, challenges and innovation in primary health care revitalization and universal health coverage implementation among 18 countries in the WHO African Region. Pan Afr Med J.

